# Variation of the epidemiological structure of thyroid cancer between year 2000 and 2012

**DOI:** 10.1186/1756-6614-6-S2-A30

**Published:** 2013-04-05

**Authors:** Aldona Kowalska, Jacek Sygut, Janusz Słuszniak, A Walczyk, Iwona Pałyga, Danuta Gąsior-Perczak, K Gadawska-Juszczyk, D Szyska-Skrobot, T Trybek, S Hurej, Ryszard Mężyk, Stanisław Góźdź

**Affiliations:** 1 Holycross Cancer Centre in Kielce, Poland

## Introduction

The incidence of thyroid cancer (TC) is increasing constantly. A standardized incidence rate in 2010 was 6.7 for women, 1.5 for men. The observed situation seems to be affected by the improvement of thyrological diagnosis and cancer detection.

Purpose of the study was to analyze the histological type and the diameter of TC and their variation over a twelve-year observation.

## Material

The study involved 1158 patients with newly diagnosed TC in the years 2000 to 2012, treated in Holycross Cancer Centre in Kielce.

## Methods

In all cases, the histological types and the diameter of cancer lesions were examined over the years. The statistic analysis of records was based on Chi-square test, Kruskal Wallis test, with the use of Med-Calc 12.4.

## Results

Histological types of TC in the entire group: papillary - 87.1%; follicular - 3%; oxyphilic -1.2%; medullary - 4.9%; poorly differentiated - 1.9%; anaplastic - 1.6%: lymphoma - 0.3%. The median of cancer diameter was 19.3 mm in year 2000 and 8.0 mm in year 2012. A statistically significant decrease in diameter of diagnosed cancer was observed over years (p<0.0001).

## Conclusions

1. Papillary thyroid carcinoma (PTC) is definitely the most frequent type of TC.

2.The difference in the percentage of different cancer types in the following years was observed due to the increase of the incidence of PTC.

3.The diameter of majority of recently diagnosed cancers is less than 10 mm.

